# Micromechanical Force Sensor Using the Stress–Impedance Effect of Soft Magnetic FeCuNbSiB

**DOI:** 10.3390/s21227578

**Published:** 2021-11-15

**Authors:** Joerg Froemel, Gildas Diguet, Masanori Muroyama

**Affiliations:** 1 Division for the Establishment of Frontier Sciences, Organization for Advanced Studies, Tohoku University, 2-1-1 Katahira, Aoba-ku, Sendai 980-8577, Japan; gildas.diguet.d4@tohoku.ac.jp (G.D.); muroyama@tohoku.ac.jp (M.M.); 2 Advanced Institute for Materials Research, Tohoku University, 2-1-1 Katahira, Aoba-ku, Sendai 980-8577, Japan; 3 Department of Robotics, Graduate School of Engineering, Tohoku University, 6-6-01, Aza Aoba, Aramaki Aoba-ku, Sendai 980-8579, Japan; 4 Department of Electrical and Electronic Engineering, Faculty of Engineering, Tohoku Institute of Technology, Yagiyama Kasumi-cho 35-1, Taihaku-ku, Sendai 982-8577, Japan

**Keywords:** magnetic thin film, force sensing, stress–impedance effect, microdevice

## Abstract

By using the stress–impedance (SI) effect of a soft magnetic amorphous FeCuNbSiB alloy, a micromachined force sensor was fabricated and characterized. The alloy was used as a sputtered thin film of 500 nm thickness. To clarify the SI effect in the used material as a thin film, its magnetic and mechanical properties were first investigated. The stress dependence of the magnetic permeability was shown to be caused by the used transducer effect. The sputtered thin film also exhibited a large yield strength of 983 GPa. Even though the fabrication technology for the device is very simple, characterization revealed a gauge factor (GF) of 756, which is several times larger than that achieved with conventional transducer effects, such as the piezoresistive effect. The fabricated device shows great application potential as a tactile sensor.

## 1. Introduction

Force sensors based on several transducer principles, such as capacitive, piezoresistive, piezoelectric, and optical principles, have been studied [1].

Capacitive-type sensors have two electric plates and a dielectric region in between. When applying force, the gap between the plates may change, which means that the capacitance may change depending on the applied force. With multiple plates, both normal forces and shear forces can be sensed. Highly elastic materials, and small-gap or large-sensor plates, can realize high sensitivity. Capacitive-type sensors are commonly used for robot applications. In [2,3], with microelectromechanical systems (MEMS) technology, the authors fabricated capacitive-type force sensor devices that can detect normal and shear forces by using multiple sensor plates.

The piezoresistive sensor uses the piezoresistive effect, which is the electrical resistivity change in a semiconductor or metal when applying mechanical strain. Based on piezoresistive materials, several types of force sensors have been developed: force sensing resistors (FSR) [4], pressure-sensitive conductive rubber, and piezoresistive fabric. FSR-based force sensors are widely used in many devices, such as joysticks. The conductive rubber is composed of a non-conductive elastomer and electrically conductive particles.

The piezoelectric effect is the ability of certain materials to generate an electric charge in response to applied mechanical stress. These materials include some quartz crystals, ceramics, and polymers, such as polyvinylidene fluoride (PVDF) [5]. Piezoelectric materials can be used for dynamic measurement only.

Conventional optical sensors use infrared light-emitting diode (LED)-based arrays and corresponding photo detectors. This sensor utilizes a light intensity change that is proportional to the strength of the pressure [6]. In [7], the authors developed a three-axis force sensor based on a CCD camera, silicon rubber, a light source, and an optical fiber-scope.

In addition to the mentioned transducer principles for force sensing in microdevices, there is also the stress–impedance (SI) effect. This effect describes the relation between the mechanical stress and the electric impedance of a ferromagnetic material. It is essentially the interaction of the inverse magnetostrictive effect, also known as the Villari effect, and the skin effect. The Villari effect is the dependence of the permeability on the change in magnetic anisotropy caused by applied mechanical stress [8,9]. The change in permeability results in a change in alternating current (AC) impedance *Z*. An electric field induced by an alternating magnetic field, caused by an AC, results in an eddy current in a conductor. Therefore, the current flow in the center of the conductor is dampened, whereas the current flow towards the edge (skin) is increased [10]. The current density *J* is expressed by:

(1) $$J=J_{S}e^{-\left(1+j\right)\frac{z}{\delta }}$$

where *J_S_* is the current density at the surface of the conductor, *z* is the distance from the surface, and *δ* is the skin depth. Δ is defined as:

(2) $$\delta =\sqrt{\frac{2\rho }{\omega \mu }}$$

where *ρ* is the resistivity and *ω* is the angular frequency of the current. The permeability of the conductor material is *µ.* When the permeability is low, this results in a large skin depth, leading to low impedance, whereas high permeability leads to high impedance. 

To describe the SI effect, several models have been developed [11]. Nevertheless, there is still no complete quantitative model that explains the function of permeability on mechanical stress. One solution was published in [12].

(3) $$H_{eff}=H+\alpha M+\frac{3}{2}\frac{\lambda_{S}}{\mu_{0}M_{S}}\frac{M}{M_{S}}\sigma$$

The effective magnetic field in the material *H*_eff_ consists of three contributions: *H*, the external magnetic field, and the magnetization (*M*)*. M* is multiplied by a dimensionless parameter representing interdomain coupling *α*. The third contribution depends on stress *σ*. It is likely that the saturation magnetostriction *λ*_S_ and saturation magnetization *M_S_* also depend on stress [13]. This model assumes that the stress acts in a similar way to an external magnetic field. Therefore, the SI effect shares similarities with the magneto-impedance (MI) effect [14,15,16,17]. 

The SI effect has been used to create strain gauges based on wires, ribbons, and thin films in the past [18,19,20,21,22,23,24]. A common parameter to describe the transducer effect is the gauge factor *GF*:

(4) $$GF=\frac{\frac{\Delta Z}{Z}}{\varepsilon }$$

where ∆*Z* is the impedance change induced by strain *ε*. A GF of more than 1000 has been reported in some studies [18]. Most of the investigated devices are rather large in size. Although applications other than as a strain gauge (e.g., for structural health monitoring, pressure sensors, or force sensors) have been proposed in the past [25,26], recently, a practical, small pressure sensing device made entirely using microtechnologies was presented for the first time to demonstrate the feasibility of MEMS sensors using the SI effect [27]. A GF of 385.7 was reported. This is larger than that of other conventionally used effects in MEMS devices, such as the piezoresistive effect of silicon.

As a next step here, based on these results, we present a MEMS force sensor with very high performance but simple fabrication technology, also using the SI effect. A key component of such devices is the ferromagnetic material exhibiting the SI effect. Often, FeSiB or CoSiB is used in investigations [28,29]. These materials are derived from the amorphous Fe_80_B_20_, which has a relatively large magnetic saturation *M_S_* at room temperature. A small substitution of the boron by silicon maintains the high magnetization while decreasing the coercive field (*Hc*). This ternary alloy was reported to be more ductile, and thus the preparation of ribbons or wires is easier. Another interesting factor of magnetic amorphous alloys is their large achievable permeability, as demonstrated in the work performed by Herzer on amorphous FeSiB, FeCoB [30], and nanocrystalline alloys [31]. This large permeability is favorable for magneto-impedance and stress–impedance transducers. Many studies have been performed on the application of this material for the magneto-impedance effect, where an applied external magnetic field reduces the permeability as the material reaches magnetic saturation. This decrease in permeability induced a change in the skin depth, as in Equation (2). As a result, a change in impedance was measured. Weak magnetostrictive materials provide the best results [32]. The magnetostrictive saturation coefficient λ_S_ can be adjusted by changing the material composition and structure. In an amorphous alloy, the iron content will result in a positive magnetostrictive coefficient, while cobalt will result in a negative magnetostrictive coefficient. The total λ_S_ can be selected by the ratio of iron to cobalt [33]. Instead of chemical substitution by cobalt, nanocrystalline αFeSi can be used for the same function. It can be obtained by annealing Fe-based amorphous alloys that contain Si, such as Fe_73.5_Cu_1_Nb_3_Si_16.5_B_6_ [34]. By adding Cu and Nd, the grain growth is suppressed. Large grains reduce the permeability according to the Herzer model. In this study, we tested and applied Fe_73.5_Cu_1_Nb_3_Si_16.5_B_6_ for the mechanical sensing of the SI effect.

## 2. Materials and Methods

To better understand the transducer material, its magnetic and mechanic properties were investigated. 

### 2.1. Investigation of the Properties of FeCuNbSiB

**Figure 1:**
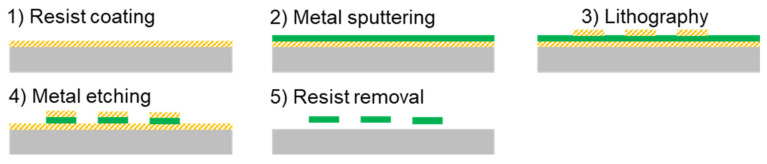
Fabrication technology to create free-standing samples of FeCuNbSiB by sputtering on resist.

To measure the mechanical properties of the investigated transducer material, a tensile test was used. For this purpose, free-standing specimens of the FeCuNbSiB alloy were made using a lift-off technique (Figure 1) in which the metallic film was sputtered onto an organic photoresist (OFPR-800, Tokyo Ohka Kogyo, Kawasaki, Japan). 

**Figure 2:**
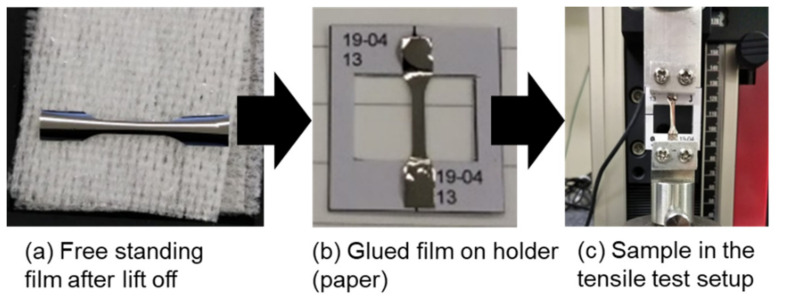
Tensile test sample: (**a**) after the fabrication process, (**b**) glued to a paper frame for handling, and (**c**) clamped into tensile test equipment.

The equipment used was a magnetron sputter with a load-lock chamber (ULVAC, QAM-4-S). The sputter power was 150 W. The substrate was rotated at 10 rpm. The deposition rate of the obtained film was 5.2 nm/min and the measurement showed a composition of Fe_78.2_Cu_1.5_Nb_2.2_Si_13.7_B_4.3_. The layer was structured by etching, and when the photoresist was removed, the samples became free. Although the thickness of the functional layer of the sensor device was planned as 0.5 µm, we chose for this experiment a thickness of 1 µm for better mechanical stability of the samples. The obtained samples (2 cm × 2–4 mm × 1 µm) were glued to a paper frame to allow for easier handling. The paper frame with the metallic film was clamped into commercial tensile test equipment (ZWICK, zwickiLine Z0.5). Figure 2 shows the sample and how it was placed into the tensile tester. After cutting the frame, a strain was applied to the film with a speed of 0.5 µm/min. The resulting force was measured and the strain and stress were calculated. The sample was strained until it broke. 

For the additional purpose of investigating the mechanical hysteresis behavior of the FeCuNbSiB thin film, a cyclic test was performed. An identical sample was placed into the same setup. Instead of straining it one time until breaking, the sample was strained until 0.8 N was reached and then the strain was decreased at the same speed until 0.1 N. This cycle was repeated 50 times, and the strain and stress were calculated and recorded.

FeCuNbSiB thin films were sputtered on 300-µm-thick Si wafers with 1 µm SiO_2_. The sputter process was the same as previously described. A titanium layer of 20 nm thickness was also used for better adhesion between the wafer and film. The film thickness was controlled by the deposition time. Samples were produced with different Ar pressures, affecting the residual stress inside the films. Samples with different stresses were made for characterization. The stress of the sputtered layers was calculated by comparing the wafer bow before and after sputtering using Stoney’s equation [35]. For the magnetic properties, samples were tested in VSM BHV-50H from Riken Denshi for measuring the in-plane magnetization curve between ±1 kOe (~80 kA/m). A Ni sample (2 cm × 2 cm × 0.1 mm) was first measured to adjust the equipment settings to match with the theoretical magnetic moment of 19.296 emu of the Ni calibration sample. After this calibration procedure, the magnetization loops of the different FeCuNbSiB samples were measured within the same range of the applied field. The magnetization was measured parallel to the film along two arbitrarily defined perpendicular axes to detect any anisotropy. Images of the magnetic domain structure were acquired by a Magnetic Optical Kerr Effect (MOKE) microscope using the in-plane mode.

### 2.2. Fabrication of the Force Sensor Using FeCuNbSiB as Transducer

The investigated material transduces mechanical strain/stress into a change in electrical impedance. A simple structure was designed to measure force based on strain/stress change. 

**Figure 3:**
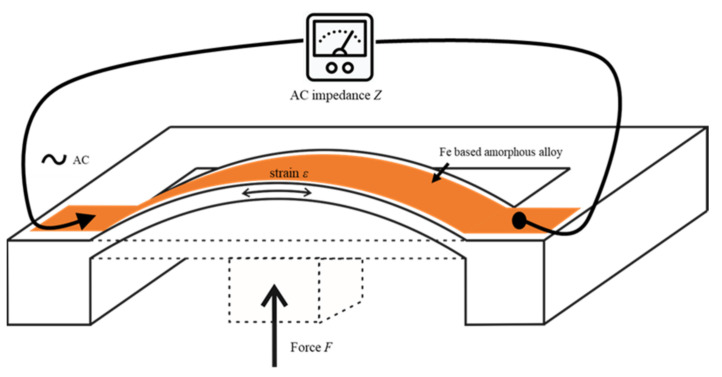
Basic structure and principle of the SI force sensor: an incoming force strains a layer of FeCuNbSiB soft magnetic alloy, which is deposited on a cantilever. The resulting change in AC impedance is measured.

Figure 3 shows the basic design and principle of the investigated SI force sensor. A double-clamped cantilever carried a thin film of soft magnetic alloy. The cantilever was reinforced in the center by a boss. A force that is applied to the boss bends the cantilever. The layer of soft magnetic alloy is strained by the bending. The resulting change in the magnetic domain structure alters the AC impedance. By measuring the AC impedance, it is possible to understand the force that is deforming the cantilever. The measurement range and mechanical sensitivity of the sensor structure can be influenced by the proper design of the length, width, and thickness of the cantilever. The soft magnetic layer on the beam was not structured to show that, by using the SI effect, very simple designs can also be used with good results. 

**Figure 4:**
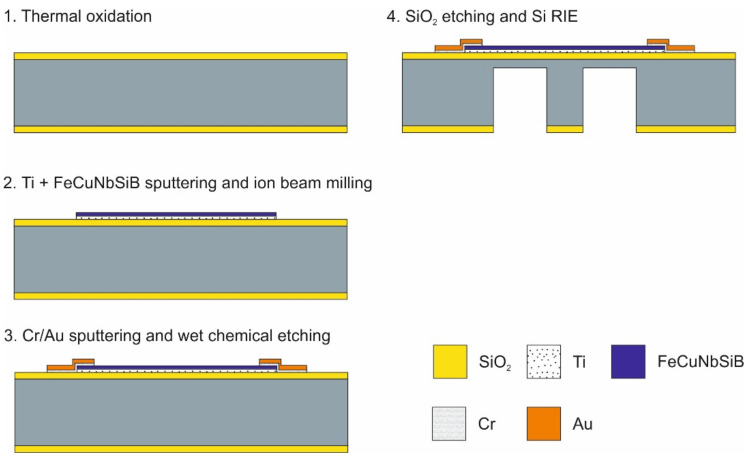
Process flow of the fabrication of the force sensor—shown by its cross-section.

The fabrication technology (Figure 4) of the proposed device is relatively simple and is described in the following section. 

A 500-µm-thick (100 mm diameter) double-sided polished Si wafer was used as a substrate. First, a 1-µm-thick thermal SiO_2_ layer was deposited on both sides. The purpose of this layer was electrical isolation, as well as creating a hard mask for silicon backside etching. After depositing an adhesion layer of 20 nm Ti, a functional layer of FeCuNbSiB was formed with a thickness of 500 nm by sputtering. To control the intrinsic stress of the non-deflected sensor structure as much as possible, the sputter process was optimized. The stress of a sputtered thin film depends on several parameters, such as substrate temperature, film thickness, sputter rate, and gas pressure. In this case, the Ar flow rate, as a way to influence the gas pressure, was chosen as the optimization parameter, and all the other conditions were left unchanged. 

**Figure 5:**
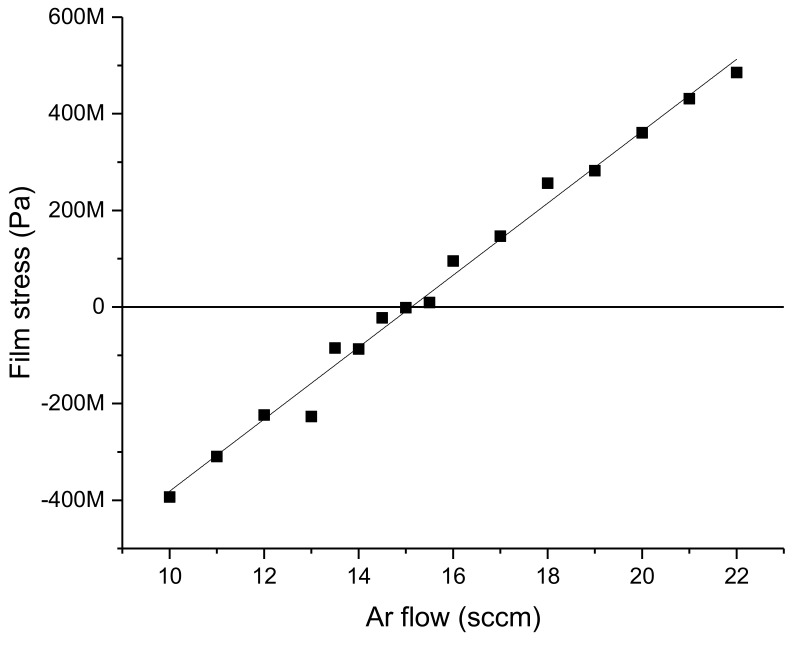
Intrinsic stress of the sputtered FeCuNbSiB against the Ar flow rate during sputtering.

In the investigated range of Ar gas flow between 10 and 22 sccm, the resulting intrinsic stress of the deposited metal layer followed an approximate linear dependence (Figure 5). In this range, stable deposition was possible. At a gas flow rate of 15 sccm, the intrinsic stress was close to 0 Pa. To confirm the structure of the film, X-ray diffraction (XRD) was used. 

The fabrication process was similar to the process described in [27]. The structuring of the metal alloy was performed by ion-beam milling using OFPR-800 200cp (Tokyo Ohka Kogyo). For the wire bonding contacts, electrodes were deposited by RF magnetron sputtering as a 400 nm Au layer and a 20 nm Cr adhesion layer. After photolithography, patterning by wet etching using aqueous iodine solution (KI + I2) was performed to form the wire bonding pads. To form a mask for the Si reactive ion etching (RIE), the SiO_2_ layer on the backside was etched by buffered HF. Through RIE (MUC-21, SPT), the cantilever was formed with a thickness of 100 µm. 

After the fabrication process, the FeCuNbSiB layer was checked by XRD. No change was observed, indicating that the material remained in an amorphous state.

**Figure 6:**
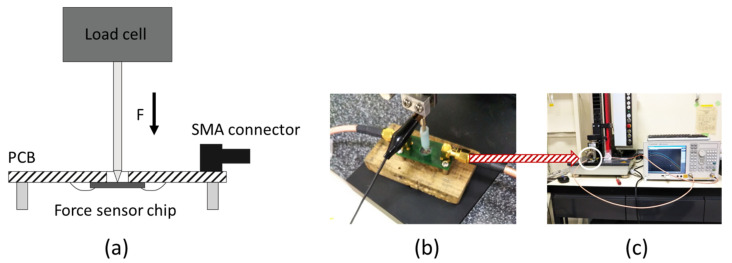
(**a**) Schematic characterization setup for the force sensor. (**b**) Picture of the PCB in the measurement setup. (**c**) Picture of the whole measurement setup, including the connected network analyzer.

The finished sensor was mounted onto a carrier-printed circuit board (PCB) and electrical contacts were made by ball-wedge wire bonding. To analyze the impedance of the sensor, a network analyzer (E5071C, Agilent, Santa Clara, CA, USA) was used. A setup to transmit a defined force to the sensor was built (Figure 6). 

To obtain the impedance of the force sensor, the S11 parameter in shunt configuration was determined by the network analyzer during the quasi-static application of defined force values. The S11 parameter results were converted into impedance values. S parameter measurements are a method of determining the behavior of circuits at high frequencies [36,37].

## 3. Results

### 3.1. Investigation of the Properties of FeCuNbSiB

**Figure 7:**
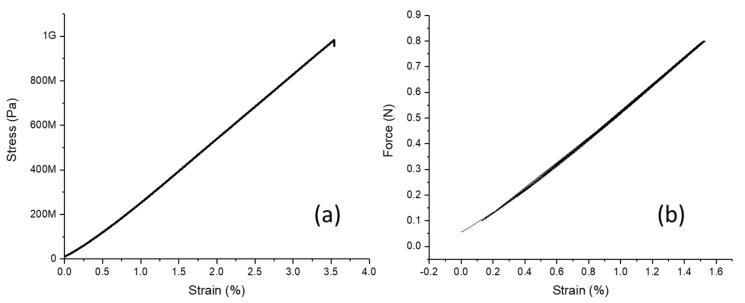
(**a**) Stress–strain curve of a free-standing sample of FeCuNbSiB alloy until breaking. (**b**) Continuously plotted force strain curve of a free-standing sample of FeCuNbSiB alloy that underwent 50 cycles of force applications between 0.1 and 0.8 N.

As a result of the tensile test of the free-standing thin films of FeCuNbSiB, it was confirmed that the material shows almost fully elastic behavior until breaking. Furthermore, the achieved tensile strength was 983 MPa, and the Young’s modulus was calculated to be 30 GPa. Even after 50 cycles of force application, there was no visible mechanical hysteresis (Figure 7). 

**Figure 8:**
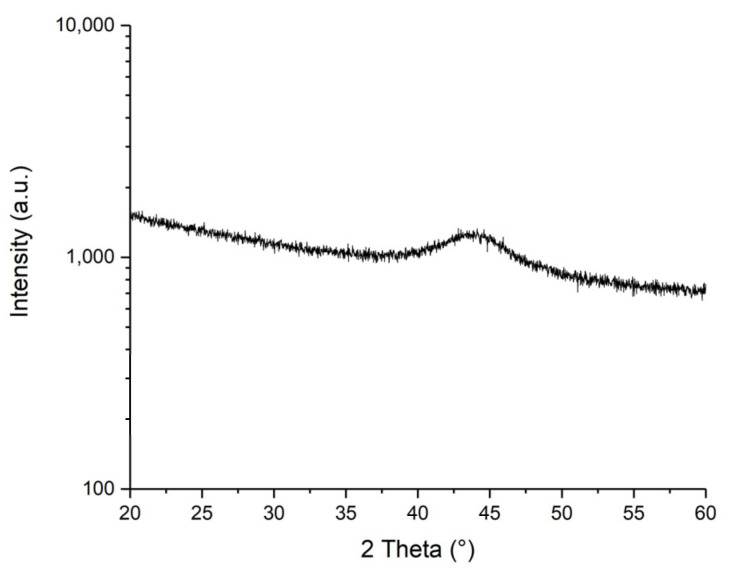
Result of the XRD measurement of the sputtered FeCuNbSiB, indicating its amorphous nature.

The structure of the sputtered material was confirmed to be amorphous by X-ray diffraction (XRD) (Figure 8).

**Figure 9:**
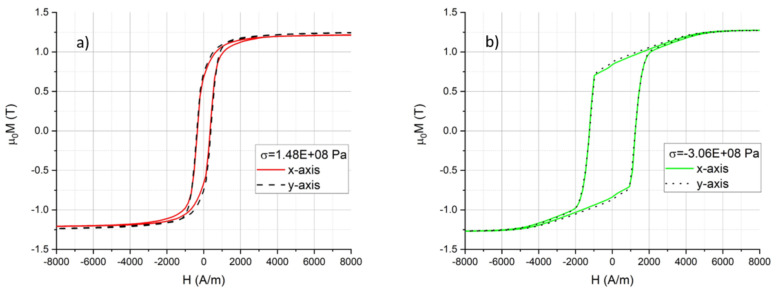
(**a**) Magnetization curves for a sample with tensile stress and (**b**) compressive stress measured along the two perpendicular axes.

The magnetization loops of samples with different thin film stresses between −400 and 500 MPa were measured according to the described procedure. An example of the result is presented in Figure 9.

**Figure 10:**
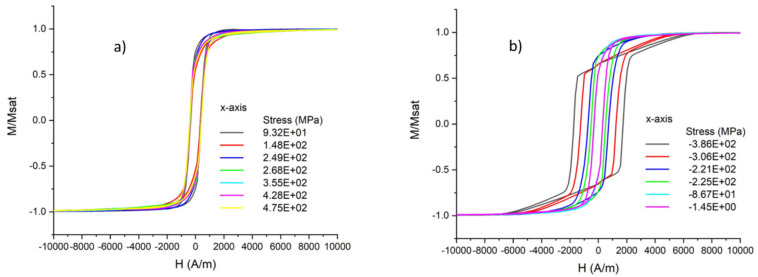
(**a**) Magnetization curves for samples with tensile stress and (**b**) compressive stress measured along the x-axis.

**Figure 11:**
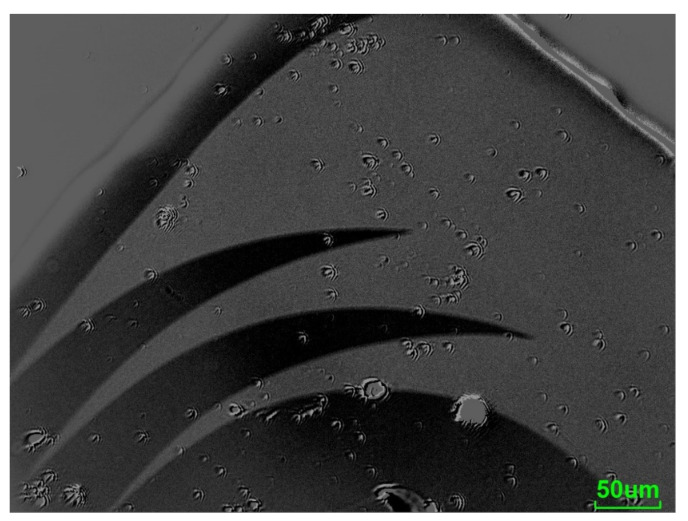
MOKE image with the sensitivity in plane at one corner of the sample with a residual stress of around −400 MPa.

It was observed that the saturation magnetization apparently does not depend on the film stress. The value of *µ*_0_*M_s_* = 1.25 ± 0.03 T was extracted from the VSM measurements. It is also visible in Figure 9 that the sputtered films did not show any particular in-plane anisotropy for all investigated stress values. The curves measured along the x-axis and along the y-axis overlapped in the compressive stress region, as well as in the tensile stress region. There was a distinct difference in the shape of the magnetization curves when comparing samples with tensile stress (Figure 9a) vs. samples with compressive stress (Figure 9b). All the samples with tensile stress showed a typical S-shaped curve (Figure 10a), whereas the samples with compressive stress (Figure 10b) had a distinctive shape with parts of a clearly different slope. Moreover, in the samples with compressive stress, the coercive field was much larger compared to that of the tensile stress region. For this set of samples, wide domains were observed in the thin film surface, with some smaller domains close to the edges (Figure 11). 

**Figure 12:**
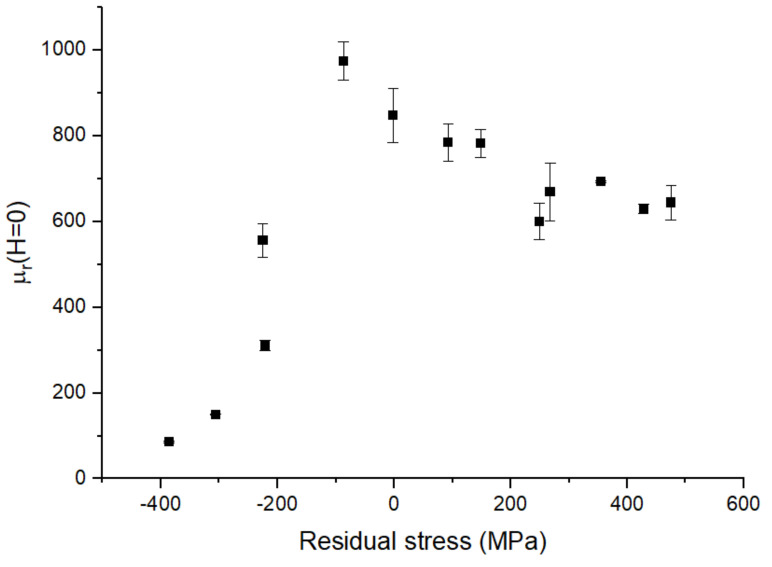
Relative magnetic permeability *µ*_r_ extracted from the magnetization curves of sputtered FeCuNbSiB as a function of the film stress.

To obtain the values for the magnetic permeability, the slope at *H* = 0 A/m was extracted from the measurement. In Figure 12, the results are shown. The largest value was observed near zero stress. From this peak, the permeability slowly decreased in the direction of increasing tensile stress, possibly approaching a constant value. On the other hand, increasing compressive stress leads to a sharp decrease in *µ*_r_, quickly approaching 1. 

### 3.2. Characterization of the SI Force Sensor

**Figure 13:**
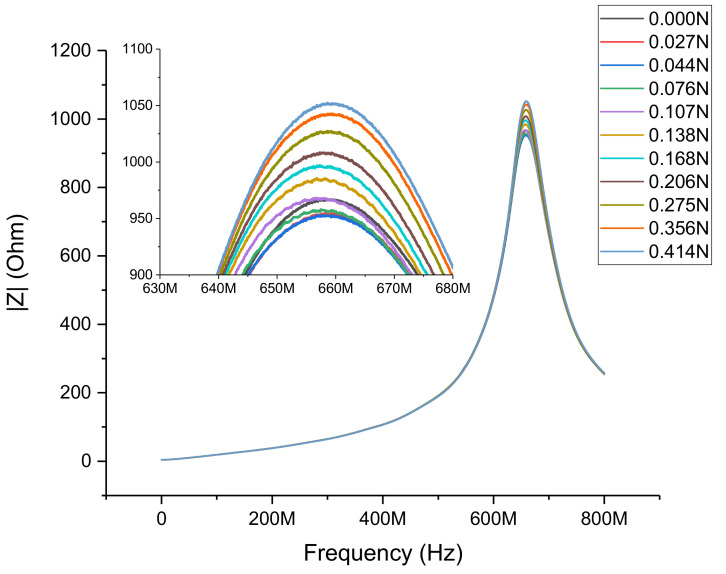
Absolute impedance of the force sensor vs. measurement frequency at some selected force loads. The inset shows a more detailed view of the resonance.

Figure 13 shows the electric impedance of the sensor versus the measurement frequency. There was a clearly visible resonance. Several curves were shown with different force loads on the sensor during the measurement. 

**Figure 14:**
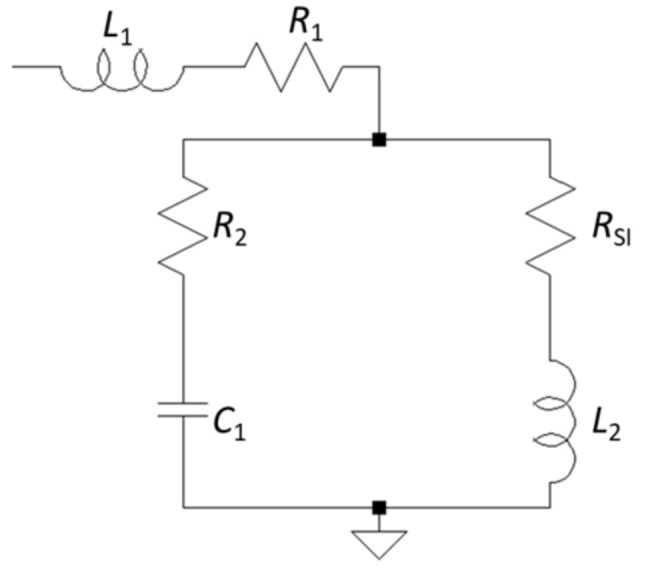
Equivalent circuit of the SI sensor device: *R*_1_, *R*_2_, *L*_1_, *L*_2_, and *C*_1_ are parasitic elements; R_SI_ is the transducer element.

In addition to the impedance of the soft magnetic sensor layer, the measured impedance of the SI force sensor also consists of several parasitic elements. This is caused by capacitive coupling through the substrate and others. A lumped sum equivalent circuit was proposed to reflect this situation (Figure 14).

The total impedance *Z* of the sensor was measured without applying any force between 1 and 800 MHz. The impedance showed a clear resonance at approximately 658 MHz. This was caused by the parasitic elements. To determine these values, we determined the error (Δ) between the measured impedance (*Z*) and the calculated impedance (*Z*_Calc_), which is given as follows:

(5) $$\Delta ={\left(Z-Z_{\mathrm{Calc}}\right)}^{2}$$

Values estimated from the dimensions of the device were used as the starting values of the fitting. The algorithm for minimizing Δ was the Levenberg–Marquart method. The results with the best fit are shown in Table 1. 

**Figure 15:**
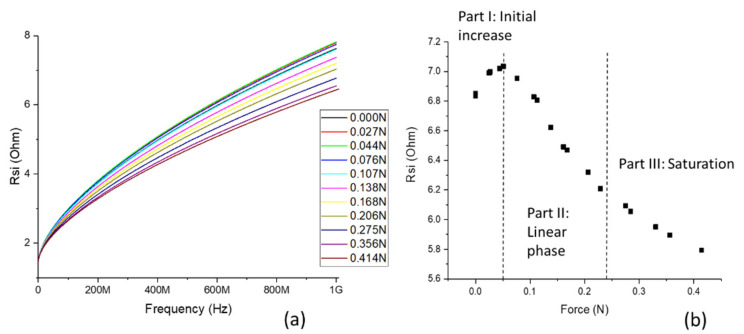
(**a**) Extracted *R*_SI_ of the force sensor vs. frequency at different force loads. (**b**) *R*_SI_ vs. applied force at 800 MHz measurement frequency.

After obtaining the parasitic elements of the equivalent circuit, the resistance of the transducer metal layer *R*_SI_ was calculated. In Figure 15a, *R*_SI_ is plotted vs. measurement frequency under different applied forces. The increase in frequency is related to the skin effect. The influence of the applied force is clearly visible. Figure 15b shows the behavior of *R*_SI_ at a frequency of 800 MHz. Three parts of the curve can be identified: Part I shows an initial increase in resistance; in Part II, the resistance decreases linearly; and in Part III, the response levels off towards a saturation value.

**Figure 16:**
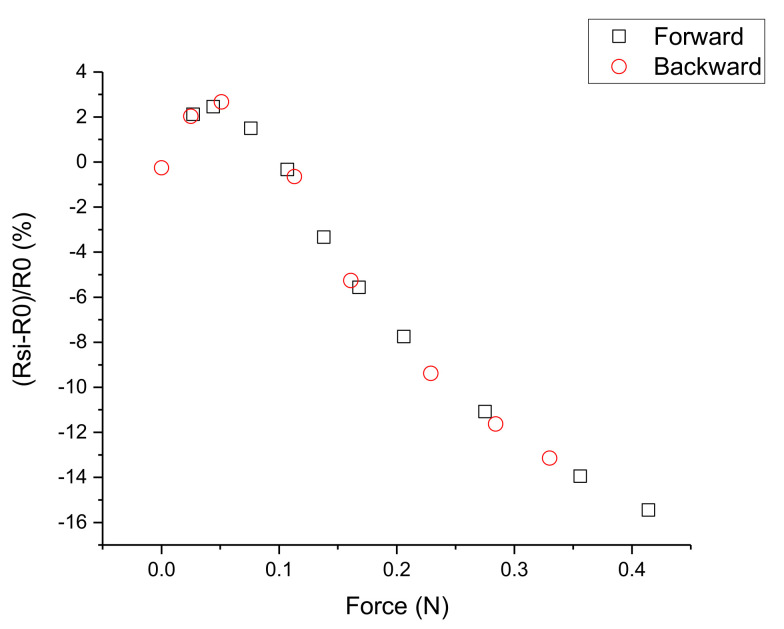
Relative change in *R*_SI_ vs. applied force. Measurement points taken in forward (force increase) and backward (force decrease) directions are shown with different symbols.

To obtain information about the hysteresis of the sensor in Figure 16, values measured in the forward (force increase) and backward (force decrease) directions are depicted. They follow the same dependence and no obvious hysteresis can be seen.

## 4. Discussion

The saturation magnetization of the sputtered FeCuNbSiB of 1.25 T is similar to the value of commercially available FeCuNbSiB sheets (Finemet) [38]. The apparent absence of an in-plane magnetic anisotropy after sputtering is explained by the sputter method. During the sputtering, the wafer rotated 10 rpm. Therefore, no preferential direction of an electromagnetic field existed. Additionally, the mechanical stress should be uniform in the x and y directions. The different behaviors of the film in the tensile and compressive stress, as depicted in Figure 9, can be explained by a difference in magnetoelastic anisotropy (*λ*_s_*σ* > 0 or *λ*_s_*σ* < 0). 

**Figure 17:**
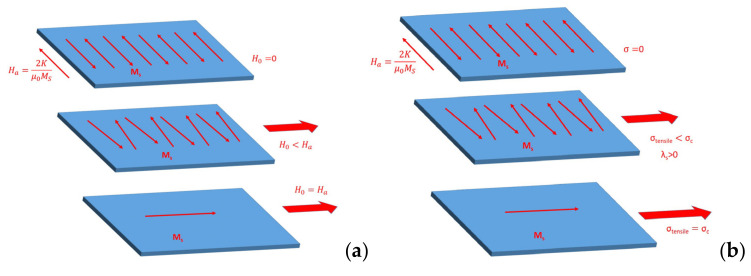
Magnetic behavior of ribbon with transverse magnetic domain with the application of a magnetic field (**a**) and a tensile stress (with *λ*s > 0) (**b**).

In the case of a thin film with magnetic domains directed along one of the two in-plane axes, as in Figure 17, the application of stress perpendicular to the domain direction (hence, the second in-plane axis of the film), the magnetization behavior is characterized by the magnetic moment’s rotation toward the applied stress direction. The resulting relative permeability (*µ*_r_ = *µ*/*µ*_0_ with *µ*_0_ = 4π10^−7^ is the permeability of vacuum) can be expressed by the equation provided by Livingston [39,40]:

(6) $$\mu_{r}=\frac{\mu_{0}M_{S}^{2}}{\left|2K-3\lambda_{s}\sigma \right|}+1$$

where *K* is the magnetic anisotropy constant. An effective anisotropy constant *K*_*eff*_ can be defined as follows:

(7) $$K_{eff}=\left|2K-3\lambda_{s}\sigma \right|$$

Equation (6) describes a case in which stress *σ* is applied perpendicularly to the magnetic anisotropy to rotate the magnetic moments in each transverse domain. This is the equivalent effect that an external magnetic field would have. When such a sample is exposed only to the applied field, this permeability is largest when the applied field *H = H*_a_ = 2*K*/*M_s_*, the so-called anisotropy field. At *H*_a_, the magnetic moments have flipped their direction. For the MI effect, the highest impedance change can be achieved at this value. The Zeeman energy (−*µ*_0_*M•H* = −*µ*_0_*MH*cos(*θ*_H_)) is minimal when the angle *θ*_H_ between the applied field and the magnetization is zero, i.e., *M* is parallel to *H*, as represented in Figure 17a. In the situation of stress–impedance (SI), stress is applied instead of a field, and the effect of stress is similar because the magnetoelastic energy −3/2*λ*_s_*σ*cos^2^(*θ*_σ_) is minimal, still assuming *λ*_s_ > 0, when the angle *θ*_σ_ between the stress and the magnetization is zero, i.e., *M* is parallel to *σ*, as represented in Figure 17b. The magnetic moments are realigned along the applied stress direction when it overcomes the anisotropy.

As shown in Equations (6) and (7), the magnetic permeability is proportional to 1/*K_eff_*. The relative permeability increases with stress, as long as the stress is smaller than a critical stress *σ*_c_:

(8) $$\sigma_{c}=\frac{2K}{3\lambda_{s}}$$

This is expected from a material with positive magnetostriction. At *σ* > *σ*_c_, the relative permeability decreases with stress. 

This model describing a system with a stress applied perpendicularly to a magnetic anisotropy is useful to qualitatively explain our results. In our measurement, biaxial stress was applied. Therefore, it was supposed that there was a component of the magnetization that was perpendicular to the applied field. Therefore, Equation (6) can basically be applied in the present case. Moreover, as the investigated FeCuNbSiB is an amorphous material, and without any magnetic annealing, the anisotropy constant *K* was replaced by a much lower (around three orders of magnitude) value <*K*> according to the random anisotropy model (RAM) [30]. This low magnetic anisotropy <*K*> is then insufficient to provide a magnetic stripe domain structure. With the very small <*K*>, the effective anisotropy constant is dominated by the magnetoelastic term 3*λ*_s_*σ* and the biaxial stress directly impacts the magnetic domain pattern. However, this model (Equation (6)) assumes a defined anisotropy direction, whereas, in our case, no difference was observed between the x- and y-axes in the magnetization loop as a consequence of the biaxial residual stress. A better description can be achieved by averaging all possible directions in-plane (from −π/2 to +π/2). This model also shows the decrease in permeability with stress [41]. 

For the compressive stress region, the situation is more complex, and the permeability is abruptly reduced with increasing stress. The shape of the magnetization loop in Figure 9b has the appearance of in-plane magnetization of a thin film that possesses an out-of-plane magnetic anisotropy. Such a shape was reported in the case of NiFe under tensile stress [42]. NiFe alloys are known to have a negative saturation magnetostriction constant depending on the Ni concentration. These alloys exhibit a negative magnetoelastic constant (~*σλ*_s_). This negative anisotropy constant then promotes normal magnetization to the film plane. The competition between this out-of-plane anisotropy and the demagnetizing anisotropy results in a magnetization direction in the plane tilted at an angle of *θ*_0_. The magnetization curve measured in the plane shows two slopes before the saturation because of the two rotations of the in-plane magnetization component and the rotation of the out-of-plane magnetization component. The obtained measurement results for the sputtered FeCuNbSiB suggest a similar case but with positive *λ*_s_ and compressive *σ*. This phenomenon greatly affects the magnetization loop, as seen in Figure 10b.

With regard to the mechanical properties, the investigated FeCuNbSiB exhibited a relatively high yield strength of close to 1 GPa, which is very high for a metallic thin film. This characteristic is known for many amorphous metallic alloys—so-called metallic glasses. For similar FeSiB alloys, the yield strength can be as high as 3.5 GPa [43], which allows device designs with large deformations that result in relatively large stress in the layer. The cyclic testing under tensile strain of a free-standing film showed no clear hysteresis in its Young’s modulus, even when it was strained by more than 1%. Although this study was limited to 50 cycles, this is an indication that the material does not change its mechanical properties by the external actuation of the device.

The next section is focused on the sensor made with the investigated soft magnetic thin film using the SI effect. The measurements of the fabricated sensor show the clear dependence of the electrical output (impedance) based on the input (force). As confirmed by material characterization, the stress applied to the sensor layer by deformation of the beam, caused by the applied force, changes the magnetic anisotropy. This is reflected in a change in the skin depth *δ* (Equation (2)), resulting in a change in impedance. In the case of the investigated sensor, there were three parts with different distinguishable behaviors (Figure 15b). As a result of the material analysis and theory, it was expected that *R*_SI_ would decrease with applied force [11]. However, at first, the value was increased. The beam of the sensor consisted of silicon and a thin layer of thermally grown silicon dioxide on one side. It is known that thermally grown silicon dioxide on silicon is under compressive stress because of the mismatch between the molar volumes of silicon and silicon dioxide. This stress resulted in a downward (in the sense of the directions in Figure 3) bending of the beam, and the transducer layer was already under some initial strain, causing compressive stress also in the transducer layer. When a force was applied, the bending—and, therefore, the stress—was reduced. The magnetic permeability increased, leading to a decrease in *δ*, which resulted in increasing impedance. When the stress became *σ*_c_, the magnetic permeability was highest, and the maximum value for *R*_SI_ was also reached. A further increase in the applied force bent the beam upwards, inducing tensile stress, and *R*_SI_ decreased, as expected. In this second phase, the *GF* as in Equation (4) could be determined, which was 756. This is significantly higher than the *GF* of conventional strain transducers, such as metal strain gauges (<10) or Si piezoresistive strain gauges (~200), and is close to values published for large-sized, wire-based sensors [18]. To reduce the problem caused by the initial compressive stress in the beam, in the future, the fabrication technology should be improved to reduce or compensate for the compressive stress. Then, the beam should be flat in the initial state and the linear interval should start at 0 N. 

## 5. Conclusions

FeCuNbSiB shows a stress-dependent change in its electric impedance (SI effect). This can be explained by the magnetoelastic theory. Because of this effect, it can be used as a transducer material. Another advantage is its relatively high mechanical strength. Despite its simple structure, the demonstrated SI force sensor, using FeCuNbSiB as a transducer material, shows high potential. It has a gauge factor several times higher than conventional transducers, indicating large sensitivity. Nevertheless, to continue research into practical applications, several points must be investigated: quantitative evaluation of hysteresis and drift behavior, the influence of perturbation (temperature, magnetic field), and compensation thereof by circuits (e.g., bridge circuits). 

For actual robotic use, the developed force sensors have large potential due to their low hysteresis and high sensitivity for sensing features, the high mechanical strength, and their ease of use for fabricating any type of sensor shape. Since force sensors are physical touch sensors, MEMS-based sensors are usually sensitive to the applied overload force. To avoid the overload, in [44], a sensor covering structure for an Si-based MEMS sensor was developed. However, the proposed sensor does not need such a protection structure due to its high mechanical strength. For accurate object recognition and handling, high-performance sensing features, including low hysteresis and high sensitivity, are important. In [44], the sensor features were used for object slip detection and texture recognition. The developed sensor can also be used for applications with the sensor features. To install a large number of force sensors, in addition to the high sensing performance described above, sensing system mechanisms, such as signal processing and network structure, should be considered. In [45,46,47], a sensor platform LSI, which has sensor readout, signal processing, and sensing data transmission circuits, was introduced. Importantly, the LSI can be integrated with several types of MEMS-based sensors. With the LSI, a tactile sensor network system was established. In [46], a 100 MEMS-LSI integrated sensor-connected tactile sensor network system was realized. However, the fabrication of the integrated device was complex and time-consuming. As the developed fabrication flow in this paper can be used for integration with the LSI, making a large network-sized tactile sensor system would be easy compared to the existing method. By using large network-sized tactile sensors, we can acquire a large number of touch sensing data with different robot operation conditions. As a result, by combining the obtained data and machine learning algorithms, we can construct accurate and reliable touch information generation systems for the future, when robots and humans will coexist.

## Figures and Tables

**Table 1:** Values of the element of the lumped equivalent circuit (Figure 14) obtained by fitting.

Lumped Equivalent Circuit Element	Fitted Value
*R*_1_	3.98 Ω
*R*_2_	5.29 Ω
*L*_1_	1.73 nH
*L*_2_	2.87 nH
*C*_1_	2.26 pF

## Data Availability

Not applicable.
